# Privacy Perceptions and Concerns in Image-Based Dietary Assessment Systems: Questionnaire-Based Study

**DOI:** 10.2196/19085

**Published:** 2020-10-15

**Authors:** Aakash Sharma, Katja P Czerwinska, Lars Brenna, Dag Johansen, Håvard D Johansen

**Affiliations:** 1 Department of Computer Science UiT - The Arctic University of Norway Tromsø Norway; 2 Faculty of Design, Computer Science, Media RheinMain University of Applied Sciences Wiesbaden Germany

**Keywords:** privacy perception, privacy, dietary assessment, mobile food records, image-based dietary assessment, data sharing, human factors, mobile phone

## Abstract

**Background:**

Complying with individual privacy perceptions is essential when processing personal information for research. Our specific research area is performance development of elite athletes, wherein nutritional aspects are important. Before adopting new automated tools that capture such data, it is crucial to understand and address the privacy concerns of the research subjects that are to be studied. Privacy as contextual integrity emphasizes understanding contextual sensitivity in an information flow. In this study, we explore privacy perceptions in image-based dietary assessments. This research field lacks empirical evidence on what will be considered as privacy violations when exploring trends in long-running studies. Prior studies have only classified images as either private or public depending on their basic content. An assessment and analysis are thus needed to prevent unwanted consequences of privacy breach and other issues perceived as sensitive when designing systems for dietary assessment by using food images.

**Objective:**

The aim of this study was to investigate common perceptions of computer systems using food images for dietary assessment. The study delves into perceived risks and data-sharing behaviors.

**Methods:**

We investigated the privacy perceptions of 105 individuals by using a web-based survey. We analyzed these perceptions along with perceived risks in sharing dietary information with third parties.

**Results:**

We found that understanding the motive behind the use of data increases its chances of sharing with a social group.

**Conclusions:**

In this study, we highlight various privacy concerns that can be addressed during the design phase. A system design that is compliant with general data protection regulations will increase participants’ and stakeholders’ trust in an image-based dietary assessment system. Innovative solutions are needed to reduce the intrusiveness of a continuous assessment. Individuals show varying behaviors for sharing metadata, as knowing what the data is being used for, increases the chance of it being shared.

## Introduction

### Background

Food images are highly relevant for use in medical research and sport science. They can capture continuous and accurate measurement of diets, and therefore are imperative in understanding the relationship between food intake and athletic development [1] or between food intake and health problems such as noncommunicable diseases [2]. The ubiquitous and increasingly capable smartphone is, in particular, becoming an essential asset that many studies now include the use of smartphones to gather data, thereby enabling new findings and development of new research methodologies [3]. The use of smartphone cameras to document meals has already been suggested as a way of improving nutrition research data and generating new insights [4-16]. By importing food pictures into an image-based dietary assessment (IBDA) system, trained professionals can go through individual dietary habits and offer personalized recommendations. Capling et al [17] surveyed the issues in dietary assessment methods in athletes and highlighted the problems of bias, accuracy, and burden on the user. IBDA systems are designed to address those issues. Thompson and Subar [8] argue that IBDA methods have the potential for research as they require less effort compared to traditional dietary assessment techniques for reaching comparable accuracy.

Although research on human subjects is already strictly regulated by local, national, and international boards and procedures, the increased usage of personal information recorded automatically through new technology comes with new concerns for the security and privacy of the subjects. Little attention has been given to the specific individual privacy requirements related to the design of IBDA systems [7,18], and how privacy awareness in larger cohorts can change over time and with regulatory discussions and coverage of privacy controversies in media [19]. For research studies based on IBDA data such as large epidemiological studies and sports science studies [1], the lack of a proper privacy framework for food-related images makes it difficult to follow Privacy by Design [20] guidelines, which recommend incorporating privacy requirements early on from the design phase, and risk not being compliant with legal and ethical laws and regulations. Participation in voluntary studies relies heavily on trust [21], and any damage to reputation can have severe consequences to organizations that obtain data based on informed consent. Thus, it is crucial to understand the privacy perceptions and concerns before implementing solutions at a population-wide scale.

To improve our understanding of the privacy requirements in population-based research data, we conducted a web-based *survey* wherein subjects were asked about their perceptions of privacy with regard to capturing food images by using a smartphone camera. Taking food pictures is already a trend on many social networks, where people typically post images of their meals during vacations and special events [22], and therefore, this is something that many can relate to. Since our general field of study is performance development of elite athletes, we selected a cohort of young participants (<35 years of age). Further, to avoid selection bias by using just a local cohort of athletes, we selected motivated cohort members from throughout the world. It is important to notice that our ongoing interdisciplinary work has involved sports science and elite athletes of several national (soccer) teams spread throughout Europe. Hence, having similar characteristics such as age, despite not elite athletes yet, in this first study, resembles this distributed target cohort. We purposely did not use the elite athlete cohort in this inaugural study owing to previous experiences of introducing new technologies to them [23,24]; our experience is that the dropout rate of such distant cohort members is way too large after the first week or so. Instead, we selected a distributed cohort that we knew would be motivated to be data contributors over a longer period.

Another important lesson from previous epidemiological-related work is that the data capturing should not be intrusive. We have previously attempted to use, for instance, 24-hour dietary recall–inspired methods with pictures taken during meals, but this showed to be too intrusive and too time-consuming for elite athletes. Moreover, the dropout rates of these elite athletes were very steep with these methods. Hence, we developed this survey by using alternative schemes for data assessment, wherein pictures of meals were captured similar to that captured in social media engagement.

From a more general perspective on privacy, food images offer an interesting case to study, as few food images might not carry much sensitive information. However, a large individual data set of images that is continuously recorded over long periods (>2 weeks) and linked to an individual’s identity might disclose information that many find too sensitive to share. Such disclosure is a growing public concern [25,26] and therefore is an interesting use case for us to explore. Our hope is that the insight gained in our survey will be useful when designing data collection projects, thereby increasing trust and compliance, which are both necessary for public engagement that most cohort studies rely so heavily upon.

### Literature Review

Systematic reviews of dietary assessment methods [17,27] have highlighted the issues of the burden on the user, accuracy of reported data, and bias in existing methods. Both studies argue about the potential of using IBDAs to address some of these concerns. Various studies [9-16] have validated the effectiveness of an IBDA system. However, they do not discuss any privacy concerns that might arise due to data collection in such tools. Furthermore, privacy risks are amplified by the increased willingness to self-disclose on one’s smartphone [28]. In this regard, Christin et al [29] studied potential privacy violations in participatory studies that collect and process sensory data recorded by mobile devices. This work investigates violations such as revealing the location by a global positioning sensor in a mobile phone and provides strategies for safeguarding privacy. Their approach only attempts to identify privacy risks linked to sensory data. Avancha et al [30] studied privacy requirements for personal health care by using mobile technology. Their extensive work investigates privacy in a mobile health (mHealth) context. They elaborately defined a conceptual framework for privacy in mHealth from legal and technological aspects. Their work also provides properties for a privacy-aware mHealth system. Some of the privacy-relevant requirements discussed in this work are inspired by their work.

Zerr et al [31] explored classifying an image as private or public based on its contents. Their work highlights preliminary research focus in this domain. They built a machine learning model from photos marked by humans as private or public. Spyromitros-Xioufis et al [32] expanded on the work of Zerr et al by using classifiers based on the content of the image by using tags (eg, erotic, alcohol, drinking). A further layer of personalization was added by training the categories that a user wants to keep private. Squicciarini et al [33] further improved the classification of Zerr et al [31]. While these approaches lay important groundwork for privacy perception on images, they do not study contextual privacy implications for a specialized purpose such as for dietary assessment. In this work, we attempt to understand the privacy implications of recording dietary intake by using images, and to the best of our knowledge, this topic has not been covered earlier.

The work on IBDA methods by Boushey et al [4,11] and O'Loughlin et al [7] mainly discuss the identification of food from images. Their work does not discuss the privacy implications of recording diets over a long period. Thomaz et al [34] investigated privacy violations while recording eating behaviors with a wearable camera. Their work tries to understand the privacy implications of using a wearable camera. The wearable camera discussed in their work takes an image at periodic intervals, which might capture other images as well. They identify privacy implications such as capturing other people’s faces or taking pictures inside a restricted location. The work further addresses these issues by offering novel solutions such as capturing images during certain hours instead of continuous captures. Similar to the work of Thomaz et al [34], Greiner and Yang [35] investigated issues with continuous recording by using a wearable camera for dietary assessments in obesity studies. They recommend postprocessing of the captured video in order to avoid any privacy violations. In our work, we do not target a wearable camera. Rather, we investigate the privacy implications of recording diets by taking images of food by individuals in a continuous study. We define a continuous study as taking pictures of food over a period of time instead of taking pictures periodically as in the study by Thomaz et al [34]. We build upon the fact that individuals are already taking selective pictures of food during vacations and sharing them on social networks [22]. Individuals are often not aware of their privacy being exposed by their data [19,25]. It appears that the awareness about data use, when shared with third parties, is often overlooked.

## Methods

### Questionnaire and Ethical Approval

To improve our understanding of the privacy perception related to the capture and use of food images, we conducted a study by using a web-based questionnaire (Multimedia Appendix 1) hosted by Nettskjema, a secure web-based survey tool hosted by the University of Oslo, Norway [36]. The questionnaire was developed using close-ended questions for their statistical analyses [37]. We simplified the questions, added a probability-severity matrix, and refined our goals through multiple pretesting/run-throughs in the laboratory. Some questions are repeated in the questionnaire to reduce biased context [37]. Responses were collected between February and June 2019. Based on our institution’s research policy, we applied for ethics approval from the Norwegian Centre for Research Data. We did not collect any personally identifiable information. After a review, we obtained an exemption from the Norwegian Centre for Research Data. The full questionnaire is available in Multimedia Appendix 2 along with the collected data.

### Design

The survey was designed to record participants’ perceptions in the following scenarios: (1) Scenario A, the participant having to record and share dietary data as an athlete; (2) Scenario B, the possibility and severity of privacy leak from one’s dietary data; and (3) Scenario C, sharing dietary data and reports among different social groups.

The scenarios were designed to be familiar to our participants and to cover various angles on data sharing. For Scenario A, sharing is both internally and externally motivated and controlled by the subject; however, the subject is not in control of the processing. For Scenario B, the subject is not in control of the data processing but has concerns about the processing outcomes, and for C, the sharing is consensual but is based on external motivation from different social groups, for example, receiving feedback from a doctor or sharing with family/friends as part of social behavior/interaction.

We used these scenarios to record our participants’ perceptions and attitudes toward sharing data. Note that we considered the perceptions on a scenario valid even for participants who never encountered that scenario in real life beforehand. One’s perceptions can affect one’s participation in a study if the concerns are not addressed at the beginning of a study.

Our questionnaire starts by familiarizing participants with food pictures on social networks (Scenario A). It then asks about the use of social networks and experience with taking food pictures. Additionally, participants are asked about their preference of IBDA methods over other similarly used techniques for dietary assessment. Attitudes toward privacy and personal control over data were collected on a 5-level *Likert* item, ranging from *strongly disagree* to *strongly agree*. These include perceptions toward responsibility for privacy, intrusiveness, and general attitude toward dietary practices [22].

Next, the participants are asked to consider Scenario A—an athlete who records his/her diet by taking pictures. We specify that every meal is recorded by taking a picture, including drinks, and even at events outside training by using a mobile app. We specify that the app allows the team owner, manager, coach, and doctor to monitor his/her diet and recommend diet plans. Continuing with questions from Scenario A, we further obtain responses toward the privacy and usability of metadata collected through such a system. Table 1 shows an excerpt of the questionnaire. For the complete questionnaire, refer to the Multimedia Appendix 2.

**Table 1:** Few questions from the questionnaire with their possible responses.

Question category, symbol		Question text	Response
**Social media usage**			
	G1	Do you use any social media platforms like Facebook, Twitter, Instagram, Snapchat, etc?	Yes/No
**IBDA^a^-related questions**			
	E1	Is taking a picture of food easier than writing down what you ate?	5-point Likert scale
	E2	Is taking a picture of food easier than recording an audio describing what you ate?	5-point Likert scale
	E3	Is it intrusive to take pictures of food every time you eat?	5-point Likert scale
	E4	Should any use of your data require an explanation in simple clear words?	5-point Likert scale
	E5	Can your doctor share your data for research with his/her colleagues without your consent?	5-point Likert scale
**Demographic questions**			
	D1	What is your educational qualification?	Level
	D2	Do you follow a religious diet?	Yes/No

^a^IBDA: image-based dietary assessment.

Regarding metadata collected through an IBDA system, we presented existing social network jargon that many are familiar with. For example, some users *tag* the location of a restaurant when posting a food picture. Further, we presented the hypothetical situation of a third party that gains control of a participant’s diet data of 1 year. Based on that data, a few aspects of the individual might be inferred. We presented Scenario B and collected responses on what the participants thought can be inferred. The inferred information examples were hypothetical, and to our knowledge, no such work exists. It was designed to evaluate perceptions toward what is possible and how sensitive particular information is to the participant. The responses were collected on a 3-item likelihood and sensitivity *Likert* scale.

In addition to perceived threats with sharing food pictures and subsequently data set, we collected responses about which social groups an individual was voluntarily willing to share information about their diet with. The information as food images and attached metadata typically associated with an image was considered for sharing. In addition to food images, we added additional parameters such as medications and diet plans. The groups provided were *Family*, *Friends*, *Doctor*, *Team*, and *Fans*. Participants indicated their binary responses by checking corresponding blocks in the questionnaire. Additionally, we provided an option if they thought the information is sensitive and they do not wish to share with anyone. At the end of the questionnaire, the participants were asked a series of demographic questions, including some additional ones about their diet and allergy. The collected data from Nettskjema were downloaded and analyzed after the end of the study. The results are presented in the Results section.

### Analysis

We performed the Kruskal-Wallis test [38] to determine the differences among responses for E1-E5 based on participants’ religious diets. Consistently, we obtained *P*>.05 supporting the hypotheses that the responses are uniform across the participants. The actual *P* values that we obtained were *P*=.14, *P*=.15, *P*=.56, *P*=.18, and *P*=.78. We performed another set of Kruskal-Wallis tests to determine whether the responses among European and non-European participants had statistically significant differences. For E1-E5, the observed *P* values, that is, *P*=.20, *P*=.14, *P*=.28, *P*=.92, and *P*=.50 indicated that they were not different. Therefore, we proceeded with reporting ordinal variables in our results by using compound bar charts.

Additionally, we measured consensus among the reported ordinal values by using the Tastle and Wierman’s consensus measure [39]. We report the consensus values for E1-E5 as Cns(E1)=0.59, Cns(E2)=0.64, Cns(E3)=0.56, Cns(E4)=0.61, and Cns(E5)=0.52. We observed a moderate amount of agreement in the reported data. For the concerns and the likelihood of them being inferred from one’s dietary data, we performed the Pearson correlation analysis. The perceived *likelihood* did not indicate a strong correlation with the *concern*. The maximum correlation coefficient we observed was between the likelihood and severity of concern for “financial status” with *r*=0.41 (*P*<.001). However, it was still a low correlation. We report our observed ordinal values later in the Results section. We did not use a prediction model in our analysis as we did not find any predictor variables to be significantly related to the outcome in our analysis (*P*>.05).

### Recruitment

Chung at al [22] showed that people are more likely to take food pictures when traveling. We leveraged this insight in combination with Goodman’s *snowball sampling* methodology [40] to recruit a varied cohort to our study. Initially, we recruited 5 individuals in different regions of the world who were traveling and hence more likely to have reflected on the use of food pictures in our scenarios. We briefed our initial cohort about the goal of our study and provided them with information about the collected data. The initial cohort was then instructed to further recruit other individuals they met throughout their travels who had personal characteristics matching the selection criteria, in accordance with Goodman’s methodology. We provided direct support for the participant that had questions about the study. In total, 105 participants responded to our questionnaire.

Of the 105 participants, 99 (94.2%) indicated having an account on a social network. Of the 99 participants, 95 (96%) reported seeing food pictures at least once over social networks. Table 2 summarizes the participants’ distribution across various factors such as age, gender, and region. Approximately 47.6% (50/105) of our participants identified themselves as male, 51.4% (54/105) as female, and 0.9% (1/105) as nonbinary. These participants were from 35 different countries around the world, spread over 6 continents. Of the 105 participants, 99 (94.3%) were younger than 35 years, which corresponds to the age up to which peak performances can be maintained by athletes [41]. We also collected information about the participants’ education levels. Approximately 90.5% (95/105) of the participants indicated that they attained an education more than high school; 44 participants indicated having higher than a bachelor’s degree (postgraduate degree, n=35; doctorate degree, n=9). Only 16.2% (17/105) of the participants were following a strict religious diet. More than one-third of the participants (39/105, 37.1%) indicated having food allergies.

**Table 2:** Demographic information of the participants (N=105).

Demographic information		Total population, N=105, n (%)	Males, n=50, n (%)	Females, n=54, n (%)	Nonbinary, n=1, n (%)
**Region**					
	Africa	4 (3.8)	0 (0)	4 (100)	0 (0)
	Asia	28 (26.7)	19 (68)	9 (32)	0 (0)
	Australia	10 (9.5)	5 (50)	5 (50)	0 (0)
	Europe	54 (51.4)	22 (41)	31 (57)	1 (2)
	North America	6 (5.7)	4 (67)	2 (33)	0 (0)
	South America	3 (2.9)	0 (0)	3 (100)	0 (0)
**Age group (years)**					
	18-25	36 (34.3)	8 (22)	28 (78)	0 (0)
	25-35	63 (60.0)	37 (59)	25 (40)	1 (2)
	35-45	4 (3.8)	4 (100)	0 (0)	0 (0)
	45-55	1 (0.9)	0 (0)	1 (100)	0 (0)
	55-65	1 (0.9)	1 (100)	0 (0)	0 (0)
**Religious diet**					
	No	88 (83.8)	40 (46)	47 (53)	1 (1)
	Yes	17 (16.2)	10 (59)	7 (41)	0 (0)
**Allergies**					
	No	65 (61.9)	38 (59)	27 (42)	0 (0)
	Yes	39 (37.1)	11 (29)	27 (69)	1 (3)
	Not indicated	1 (0.9)	1 (100)	0 (0)	0 (0)

## Results

### Overview

In this section, we discuss the perception of privacy and related attitudes based on the findings in our study. We divided our results into expectations and concerns. The expectations cover general perception toward privacy, IBDA methods, and data use. The concerns cover information that can be inferred from their dietary data. Additionally, we discuss the concerns toward exposing such information to a third party from their mobile Food Records (mFRs). Finally, we present our findings regarding the sharing of collected dietary information with different social groups.

### Expectations

We present the general expectations that participants have indicated toward IBDA methods. We also explored their attitudes toward data collection and use.

#### Effort

**Figure 1:**
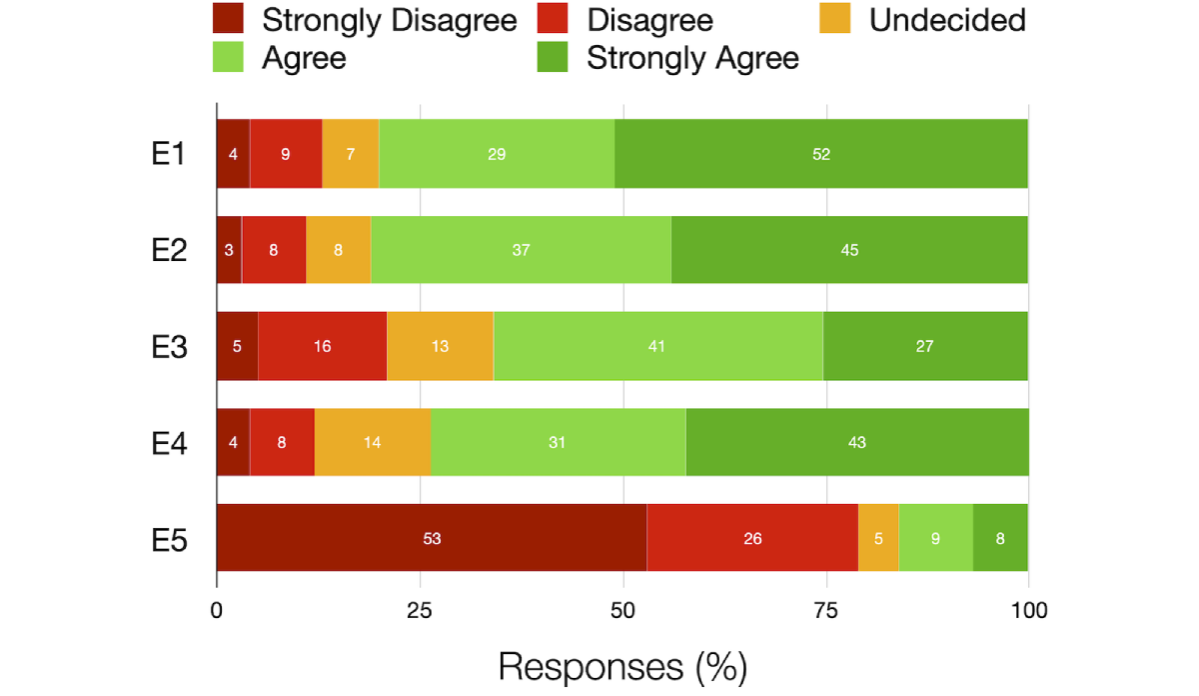
Collected responses toward E1-E5 (see Table 1). The values in the graph indicate the percentage of responses.

Approximately 80.9% (85/105) of the participants agreed that capturing diet records by using a phone camera is easier than writing down their dietary intake (see E1, Figure 1). Individuals could also record audios describing their diets for accurately recording their diets. More than four-fifths of the participants (86/105, 81.9%) preferred capturing photos over recording their diets by voice (see E2, Figure 1). Only some participants were undecided about preferring image capture over writing down or recording audio (7/105, 6.7% and 8/105, 7.6%; respectively). About half of the participants (52/105, 49.6%) had previously posted food images on social networks. We considered these participants as experienced because they are familiar with the required training for an IBDA-based study. Even those participants who lacked experience (53/105, 50.4%) in posting food images showed similar attitudes toward ease of recording their diet intakes by using photography. For a successful IBDA-based study, continuous recording of participants’ diets is required. Compared to the irregular posting of images on social networks, continuous recording requires extra effort from a participant. When we asked the intrusiveness of this requirement (see E3, Figure 1), about two-thirds of the participants (69/105, 65.7%) indicated that it would be intrusive.

#### Data Use

Individuals tend to have very little or no information about how their data are being used. Many individuals feel that they have no control [42]. In 2018, general data protection regulation (GDPR) [43,44] granted additional rights to individuals about their data in Europe. In our study, nearly half of our participants (54/105, 51.4%) were based in Europe (see Table 2). We recorded our participants’ expectations and attitudes toward data use, some of which are enforced by GDPR. Overall, three-fourths of the participants (78/105, 74.2%) wanted to know about any use of their data (see E4, Figure 1). They preferred it to be explained in simpler terms over the complex “*terms of use*.” While Europeans have a legal right to demand such explanations, we excluded data from them to see what the participants from outside Europe prefer. Even among non-Europeans (38/51, 75%), we observed a similar interest in the participants in knowing what their data are being used for.

### Concerns

Trust is important for participation in epidemiological research [21]. The early stages of newly developed methods rely heavily on voluntary participation from willing individuals. Building and maintaining trust in research is critical, especially while handling personal information. Data leaks can expose information about individuals that can be sold to third parties with potentially malicious intent. In our study, we presented a scenario in which mFR data of an athlete were leaked to a third party. We are not aware of works inferring information about an individual from their mFRs. We investigated the participants’ attitudes toward issues that might arise after their information is leaked. We discuss attitudes toward what is perceived to be exploitable and how much concerned the participants are. We collected the responses on a 3-point Likert scale.

#### Allergies

**Figure 2:**
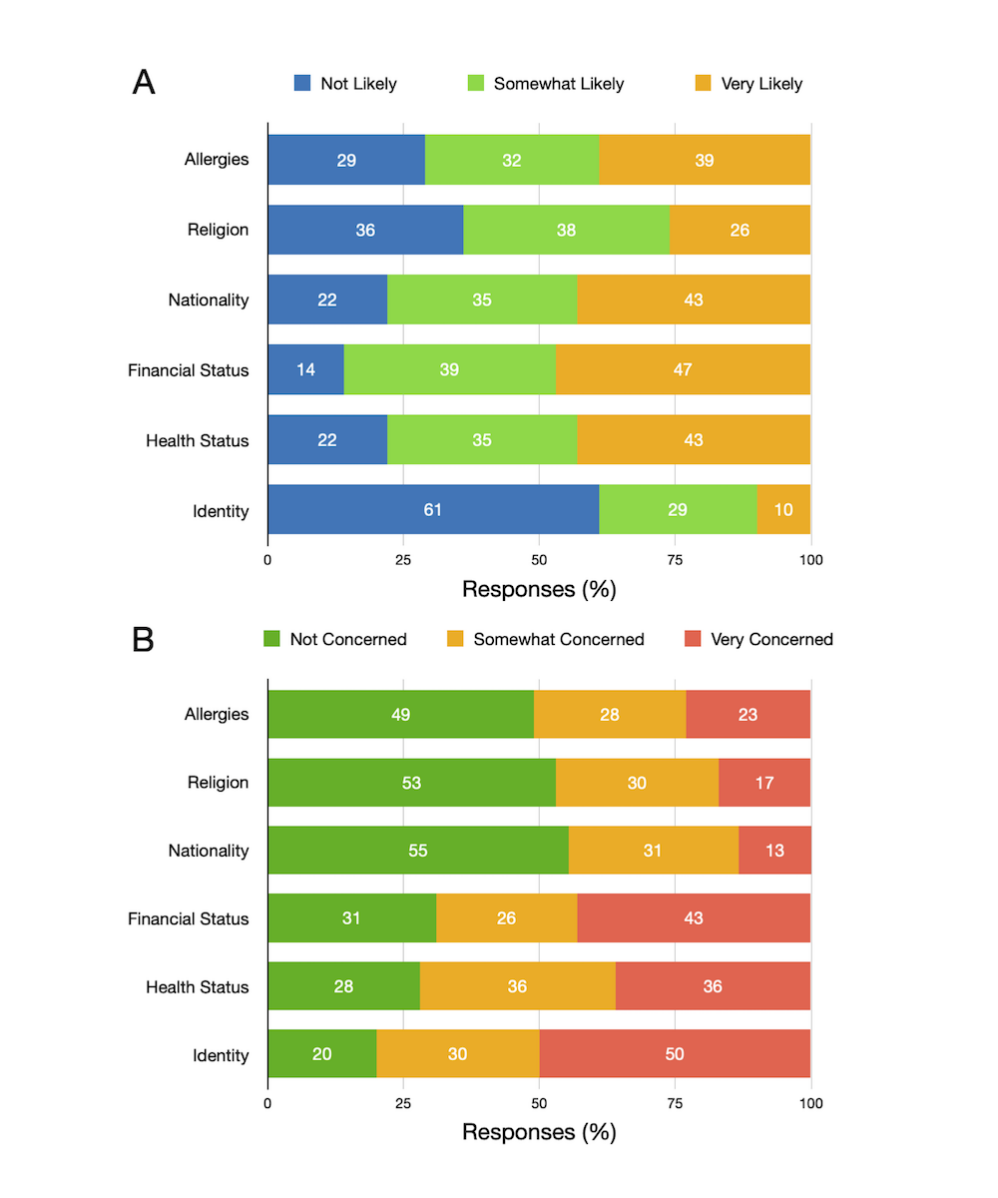
A. Perceived inference from image-based dietary assessment data set; B. Concerns toward a third party learning such attributes. The values in the graph indicate the percentage of responses.

With regard to food allergies, only a fraction of the participants (24/105, 22.8%) were very concerned about it being exposed to a third party (Figure 2). About one-third of the participants (30/105, 28.6%) were somewhat concerned. If we only consider only the participants with allergies (39/105, 37.1%), they were found to be relatively less worried. Only 13% (5/39) of the participants with allergies were particularly concerned, while 28% (11/39) indicated somewhat concerned. The majority (23/39, 59%) of the participants with allergies were not concerned about a third party learning about their allergies.

About the possibility of deriving allergies from their mFRs, more than two-thirds (75/105, 71.4%) thought that allergies can be inferred. A little more than a quarter (30/105, 28.6%) thought that it was not very likely to be derived from the mFR of an individual. This trend was very similar among participants with allergies. Approximately 30% (12/39) of the participants with allergies thought that it was not likely that it can be inferred from their mFRs.

#### Religion

Religious belief is often considered sensitive information in regional laws [43]. It can also affect the dietary choices of an individual. However, depending on the social and cultural aspects, individuals may share this information openly. In our study, about two-thirds (67/105, 63.8%) of the participants indicated that it is likely that their religion can be inferred from their mFRs. As stated earlier in Table 2, 16% (17/105) of our participants followed a strict religious diet. They showed similar traits. Three-fourths (12/17, 76%) of the participants following religious diets indicated that it is likely that their religion can be derived from their mFRs.

With regard to a third party learning about their religion, a little more than half (56/105, 53.3%) of the participants were not concerned. Only 17.1% (18/105) were *very concerned* about a third party learning about their religion. Participants following religious diets (n=17) were slightly more concerned about a third party learning about their religion. Approximately 65% (11/17) of the participants following religious diets indicated that they were concerned about a third party learning about their religion from the mFRs.

#### Identity

As personalized dietary interventions are more effective [45], IBDA systems may require personally identifiable information. Exposing the identity of an individual is one of the prominent privacy concerns in the modern era [46,47]. With regard to inferring identity from mFRs, participants of both genders showed similar attitudes. The majority (64/105, 60.9%) thought that it was not likely to infer identity from their mFRs (Figure 2). Approximately 70% (38/54) of the females and 50% (25/50) of the males responded that way. While many found it unlikely to infer one’s identity from mFRs, exposed identity was still a concern to many of the participants. In terms of gender, male participants showed a slightly higher (41/50, 82% vs 43/54, 80%) concern toward their identity being exposed to a third party when compared to females.

### Information Sharing

**Figure 3:**
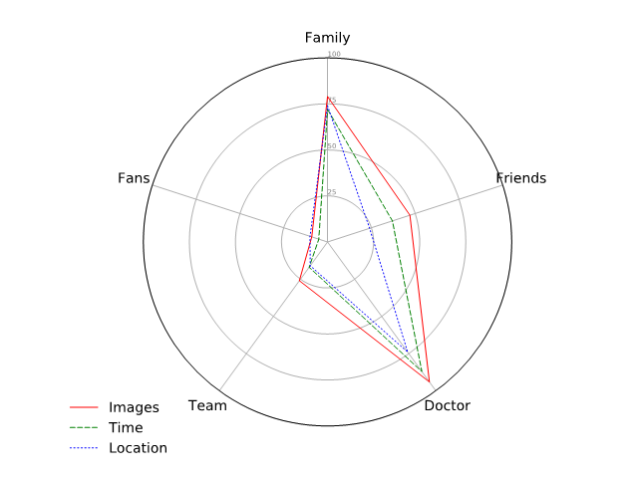
Radar plot showing willingness to share information with different social groups.

Information collected by an IBDA system about an athlete’s diet provides insights into dietary habits and can guide toward a proper diet. There might be additional metadata collection through an IBDA system. The information collected in the form of food images along with metadata can be mined for other purposes as well. For example, the time of dietary intake can be useful for maximizing performance on the field or predicting burnout. Similar to trends on social networks [22], an athlete might be interested in sharing this information with different social circles. We collected responses about sharing this information with different social groups as an athlete. The results showed that participants favored sharing information mostly with their doctors and family (see Figure 3).

About three-fourths (79/105, 75.2%) of the participants showed willingness to share food pictures continuously with family. In comparison to the social group *family*, the participants were more willing to share food images with their *doctors* (94/105, 89.5%). Only a quarter (26/105, 24.8%) of the participants showed willingness to share food pictures with their sports *team* while nearly half (47/105, 44.8%) showed willingness to share food pictures with *friends*. In terms of the metadata associated with dietary data, such as the time of the meal, the willingness to share further drops. Only 68.6% (72/105) of the participants agreed to share the *time of the meal* with their families in comparison to 82.9% (87/105) sharing the time of the meal with their doctors. Time of food intake is in fact an important consideration for elite athletes and coaches with respect to restitution and training planning.

Individuals are more cautious about sharing their location. Only 70.5% (74/105) expressed willingness to share the location associated with diet records with *family* and also with *doctor*. Unsurprisingly, participants were not very keen on sharing location with their fans (10/105, 9.5%). About 16.2% (17/105) of the participants did not want to share their location of places they eat with anyone.

**Figure 4:**
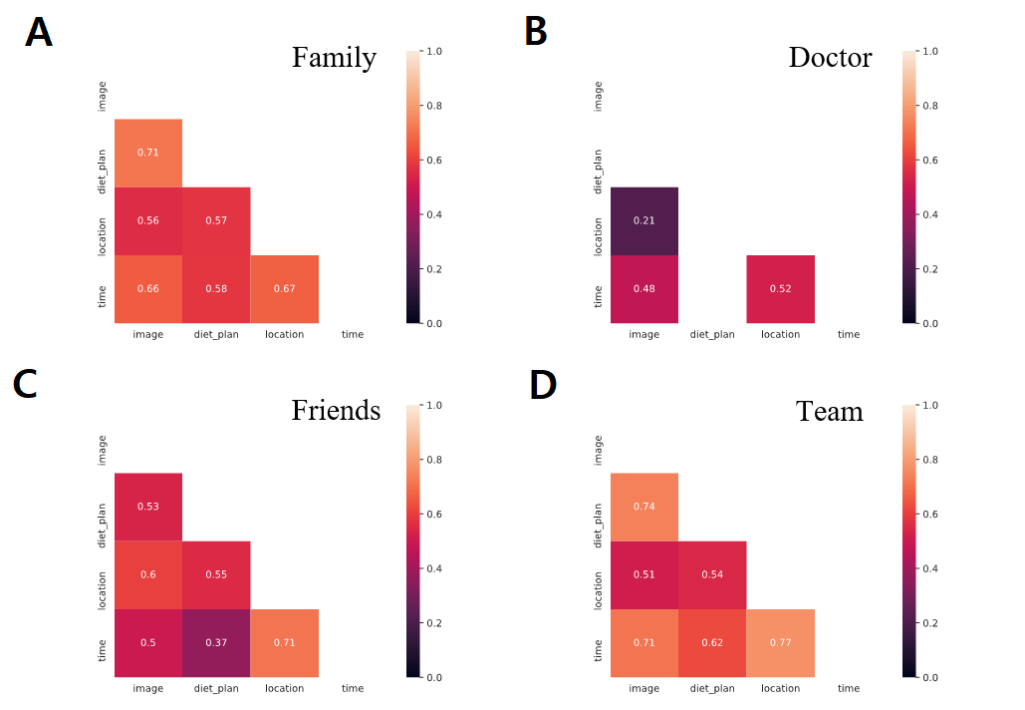
Pearson correlation values for sharing meta-information such as food image, diet plan, location and time between different social groups A. Family; B. Doctor; C. Friends; D. Team. The values indicate the correlation between the willingness, or lack thereof, to share meta-information.

Figure 4 shows the correlation values between different metadata sharing behaviors within a group. We ignored the responses from participants who indicated their unwillingness to share with anyone. All of the remaining participants agreed on sharing the food image, diet plan, and time of eating food with a *doctor*. There was a weak correlation between willingness to share location along with the food image with a *doctor.* Within the *family* group, there was a strong correlation in sharing diet plan and time. In terms of sharing metadata with one’s team, we observed a strong correlation between diet plan and food image. The willingness to share time and location with one’s team was also strongly correlated.

## Discussion

In this paper, we present our findings on the perception of privacy for an IBDA system. Our findings provide a coarse view of privacy attitudes toward conducting dietary assessments with food images. Expanding upon prior works [7,18], these results explore contextual privacy violations for an IBDA system. Trust is crucial for voluntary participation in epidemiological studies [21]. When designing such systems, following Privacy by Design guidelines is beneficial for addressing privacy concerns early on and building trust. We found that participants indicated a strong dislike toward data use without consent. Explaining data collection and processing with easy-to-understand terms seems to be of interest to users. Public engagement in cohort studies is crucial for their success. We conjecture that incorporating these parameters can deliver a pleasant experience and increase users’ trust in the system.

Personalized dietary interventions are more effective than universal recommendations [45]. However, these interventions require additional information about the user, some of which may be considered sensitive from the legal or an individual’s point of view. In this context, information such as food allergies can be acquired by third parties to improve recommendations. The additional information about a user can lead to identity leaks. Similar to previous works [46,47], identity is still a top concern for individuals interacting with web-based systems. It might be useful to prevent that in design by separating the authentication and data storage for mFRs. Accordingly, careful considerations should be taken to share inferred details about an individual, preferably only to individuals that the user has consented to share with. In this study, the participants were not worried about whether a third party could learn about their religion from their mFRs. However, regional privacy laws may restrict sharing such information [43]. These findings are not intended to replace legal requirements while building systems. They complement them to build a trustworthy system.

Our initial assumption from the study by Chung et al [22] was that individuals are willing to share food pictures over social networks. However, we experienced the opposite for long-running continuous studies. In our study, individuals preferred to share dietary data with groups who have a clear and stated use for it, such as their dietician. Another trusted group for information sharing is family. Individuals show different behaviors for sharing metadata. Thus, metadata require different sharing policies than images. For an IBDA system, this means that metadata need to be scrubbed from a food image and stored separately.

Sharing an athlete’s data with fans can be an interesting opportunity to engage with followers, such as for crowd support using HeartLink [48]. However, our study shows that individuals might refrain from sharing their data. Participants show greater willingness to share information with groups they trust or when they know what it will be used for. Motivating users to share self-reported data can be challenging. Functionality, ease of use, and privacy are considered crucial for any self-reporting health app [49]. Even chronically ill patients are willing to share data if they receive personalized feedback [49]. Such attitudes can lead to exploring use-based privacy policies for their data [50].

Elite sport clubs, particularly in our elite soccer domain, have nutritional experts hired as part of their management and support team. Such experts are involved in providing detailed dietary plans for their athletes, and they know in detail about most of the common meals provided on-premise for the athletes. For instance, our main elite soccer clubs involved in our cooperation, as a rule, have breakfast and lunch together in their training facilities. Involving such experts using our proposed scheme means that they receive the needed data from their athletes when outside the training facilities to complete the picture.

We investigated the privacy perceptions and concerns for conducting long-running studies using IBDA methods. For epidemiological studies, it is important for users to continuously record diets without any biases. In this study, individuals preferred recording diets using a digital camera over other methods. However, taking a picture of every meal is still perceived as intrusive for some users. For long-running studies, prediction models can be employed to reduce the labor of taking pictures [18]. In summary, this study provides initial insights into the privacy requirements for an IBDA system. Thus, our work provides the basis for discussion in the research community for building and deploying IBDA systems for population-wide studies.

Our study has the following limitation. A questionnaire-based study fails to identify the causation of behavior. For privacy reasons, we did not collect the contact information from the participants. Hence, any further study with the same set of participants is not possible.

In conclusion, we conducted a questionnaire-based study to understand the privacy perceptions and concerns for building IBDA systems. The privacy concerns can be addressed during the design phase to mitigate risks and strengthen participants’ and stakeholders’ trust in a system. We find a growing interest to know what the collected data are being used for. While IBDA methods are preferred for ease of use, continuous assessment is still seen as intrusive. GDPR compliance is an attractive feature for individuals worldwide. While uncertain about the inferences from mFRs, identity remains a top concern with regard to privacy for individuals. Knowing what the data is being used for, increases the chances of it being shared. Individuals are concerned about metadata sharing with third parties. We recently started a large interdisciplinary study involving computer scientists, sports scientists, psychologists, mathematicians, and medical experts (epidemiologists, nutritional scientists, physicians). Our select cohort includes over 400 female elite soccer athletes, from Norway to Portugal, and we intend to conduct our next study in this cohort.

## Appendix

Multimedia Appendix 1 The complete questionnaire from nettskjema.

Multimedia Appendix 2 The collected data from the study along with column descriptions for reproducing results. The readme and column descriptions are available in markdown format and PDF. The data file is available in TSV (tab-separated values) format.

## Abbreviations

GDPR: general data protection regulation
IBDA: image-based dietary assessment
mFR: mobile food record
mHealth: mobile health

## References

[1] Vogt M, Puntschart A, Howald H, Mueller B, Mannhart C, Gfeller-tuescher L, Mullis P, Hoppeler H (2003). Effects of Dietary Fat on Muscle Substrates, Metabolism, and Performance in Athletes. Medicine & Science in Sports & Exercise.

[2] Afshin A, Sur Pj, Fay Ka, Cornaby L, Ferrara G, Salama Js, Mullany Ec, Abate Kh, Abbafati C, Abebe Z, Afarideh M, Aggarwal A, Agrawal S, Akinyemiju T, Alahdab F, Bacha U, Bachman Vf, Badali H, Badawi A, Bensenor Im, Bernabe E, Biadgilign Skk, Biryukov Sh, Cahill Le, Carrero Jj, Cercy Km, Dandona L, Dandona R, Dang Ak, Degefa Mg, El Sayed Zaki M, Esteghamati A, Esteghamati S, Fanzo J, Farinha Cses, Farvid Ms, Farzadfar F, Feigin Vl, Fernandes Jc, Flor Ls, Foigt Na, Forouzanfar Mh, Ganji M, Geleijnse Jm, Gillum Rf, Goulart Ac, Grosso G, Guessous I, Hamidi S, Hankey Gj, Harikrishnan S, Hassen Hy, Hay Si, Hoang Cl, Horino M, Islami F, Jackson Md, James Sl, Johansson L, Jonas Jb, Kasaeian A, Khader Ys, Khalil Ia, Khang Y, Kimokoti Rw, Kokubo Y, Kumar Ga, Lallukka T, Lopez Ad, Lorkowski S, Lotufo Pa, Lozano R, Malekzadeh R, März W, Meier T, Melaku Ya, Mendoza W, Mensink Gb, Micha R, Miller Tr, Mirarefin M, Mohan V, Mokdad Ah, Mozaffarian D, Nagel G, Naghavi M, Nguyen Ct, Nixon Mr, Ong Kl, Pereira Dm, Poustchi H, Qorbani M, Rai Rk, Razo-García C, Rehm Cd, Rivera Ja, Rodríguez-Ramírez S, Roshandel G, Roth Ga, Sanabria J, Sánchez-Pimienta Tg, Sartorius B, Schmidhuber J, Schutte Ae, Sepanlou Sg, Shin M, Sorensen Rj, Springmann M, Szponar L, Thorne-Lyman Al, Thrift Ag, Touvier M, Tran Bx, Tyrovolas S, Ukwaja Kn, Ullah I, Uthman Oa, Vaezghasemi M, Vasankari Tj, Vollset Se, Vos T, Vu Gt, Vu Lg, Weiderpass E, Werdecker A, Wijeratne T, Willett Wc, Wu Jh, Xu G, Yonemoto N, Yu C, Murray Cjl (2019). Health effects of dietary risks in 195 countries, 1990–2017: a systematic analysis for the Global Burden of Disease Study 2017. The Lancet.

[3] Holmlund TB, Foltz PW, Cohen AS, Johansen HD, Sigurdsen R, Fugelli P, Bergsager D, Cheng J, Bernstein J, Rosenfeld E, Elvevåg Brita (2019). Moving psychological assessment out of the controlled laboratory setting: Practical challenges. Psychol Assess.

[4] Boushey CJ, Spoden M, Zhu FM, Delp EJ, Kerr DA (2016). New mobile methods for dietary assessment: review of image-assisted and image-based dietary assessment methods. Proc Nutr Soc.

[5] Brenna L, Johansen H D, Johansen D (2019). A Survey of Automatic Methods for Nutritional Assessment.

[6] Howes E, Boushey C, Kerr D, Tomayko E, Cluskey M (2017). Image-Based Dietary Assessment Ability of Dietetics Students and Interns. Nutrients.

[7] O'Loughlin G, Cullen SJ, McGoldrick A, O'Connor S, Blain R, O'Malley S, Warrington GD (2013). Using a wearable camera to increase the accuracy of dietary analysis. Am J Prev Med.

[8] Thompson F, Subar A F (2017). Dietary Assessment Methodology. Nutrition in the Prevention and Treatment of Disease.

[9] Myers A, Johnston N, Rathod V, Korattikara A, Gorban A N, Silberman N, Guadarrama S, Papandreou G, Huang J, Murphy K P (2015). Im2Calories: Towards an Automated Mobile Vision Food Diary.

[10] Salvador A, Drozdzal M, Giro-i-Nieto X, Romero A (2019). Inverse Cooking: Recipe Generation From Food Images.

[11] Boushey CJ, Kerr DA, Wright J, Lutes KD, Ebert DS, Delp EJ (2009). Use of technology in children's dietary assessment. Eur J Clin Nutr.

[12] de Vries JHM, de Groot LCPGM, van Staveren WA (2009). Dietary assessment in elderly people: experiences gained from studies in the Netherlands. Eur J Clin Nutr.

[13] Lazarte CE, Encinas ME, Alegre C, Granfeldt Y (2012). Validation of digital photographs, as a tool in 24-h recall, for the improvement of dietary assessment among rural populations in developing countries. Nutr J.

[14] Beltran A, Dadabhoy H, Ryan C, Dholakia R, Jia W, Baranowski J, Sun M, Baranowski T (2018). Dietary Assessment with a Wearable Camera among Children: Feasibility and Intercoder Reliability. J Acad Nutr Diet.

[15] Ashman A, Collins C, Brown L, Rae K, Rollo M (2017). Validation of a Smartphone Image-Based Dietary Assessment Method for Pregnant Women. Nutrients.

[16] Ambrosini GL, Hurworth M, Giglia R, Trapp G, Strauss P (2018). Feasibility of a commercial smartphone application for dietary assessment in epidemiological research and comparison with 24-h dietary recalls. Nutr J.

[17] Capling L, Beck K, Gifford J, Slater G, Flood V, O'Connor Helen (2017). Validity of Dietary Assessment in Athletes: A Systematic Review. Nutrients.

[18] Wang Y, He Y, Boushey CJ, Zhu F, Delp EJ (2018). Context Based Image Analysis With Application in Dietary Assessment and Evaluation. Multimed Tools Appl.

[19] Fiesler C, Hallinan B (2018). "We Are the Product": Public Reactions to Online Data Sharing and Privacy Controversies in the Media.

[20] Langheinrich M (2001). Privacy by Design — Principles of Privacy-Aware Ubiquitous Systems.

[21] Slegers C, Zion D, Glass D, Kelsall H, Fritschi L, Brown N, Loff B (2015). Why do people participate in epidemiological research?. J Bioeth Inq.

[22] Chung C, Agapie Elena, Schroeder Jessica, Mishra Sonali, Fogarty James, Munson Sean A (2017). When Personal Tracking Becomes Social: Examining the Use of Instagram for Healthy Eating. Proc SIGCHI Conf Hum Factor Comput Syst.

[23] Pettersen SA, Johansen HD, Baptista IAM, Halvorsen P, Johansen D (2018). Quantified Soccer Using Positional Data: A Case Study. Front Physiol.

[24] Thambawita V, Hicks S, Borgli H, Stensland H, Jha D, Svensen M K, Pettersen S A, Johansen D, Johansen H D, Pettersen S D, Nordvang S, Pedersen S, Gjerdrum A T, Grønli T M, Fredriksen P M, Eg R, Hansen K S, Fagernes S, Claudi C, Bjørn-Hansen A, Nguyen D T, Kupka T, Hammer H L, Jain R, Riegler M, Halvorsen P (2020). PMData: a sports logging dataset.

[25] Schroeder R (2014). Big Data and the brave new world of social media research. Big Data & Society.

[26] Graeff T, Harmon S (2002). Collecting and using personal data: consumers’ awareness and concerns. Journal of Consumer Marketing.

[27] Naska A, Lagiou A, Lagiou P (2017). Dietary assessment methods in epidemiological research: current state of the art and future prospects. F1000Res.

[28] Melumad S, Meyer R (2020). Full Disclosure: How Smartphones Enhance Consumer Self-Disclosure. Journal of Marketing.

[29] Christin D, Reinhardt A, Kanhere SS, Hollick M (2011). A survey on privacy in mobile participatory sensing applications. Journal of Systems and Software.

[30] Avancha S, Baxi A, Kotz D (2012). Privacy in mobile technology for personal healthcare. ACM Comput Surv.

[31] Zerr S, Sierdorfer S, Hare J S, Demidova E (2012). Privacy-aware image classification and search.

[32] Spyromitros-Xioufis E, Papadopoulos S, Popescu A, Kompatsiaris I (2016). Personalized Privacy-aware Image Classification.

[33] Squicciarini A, Caragea C, Balakavi R (2017). Toward Automated Online Photo Privacy. ACM Trans Web.

[34] Thomaz E, Parnami A, Bidwell J, Essa I, Abows G D (2013). Technological approaches for addressing privacy concerns when recognizing eating behaviors with wearable cameras.

[35] Greiner S (2008). Privacy Protection in an Electronic Chronicle System. http://isl.anthropomatik.kit.edu/downloads/850_NBC08_Simon.pdf.

[36] Nettskjema.

[37] Glasow P (2005). Fundamentals of survey research methodology.

[38] Kruskal WH, Wallis WA (1952). Use of Ranks in One-Criterion Variance Analysis. Journal of the American Statistical Association.

[39] Tastle W, Wierman Mj (2007). Consensus and dissention: A measure of ordinal dispersion. International Journal of Approximate Reasoning.

[40] Goodman LA (1961). Snowball Sampling. Ann Math Statist.

[41] Allen SV, Hopkins WG (2015). Age of Peak Competitive Performance of Elite Athletes: A Systematic Review. Sports Med.

[42] Shklovski I, Mainwaring S D, Skúladóttir H H, Borgthorsson H (2014). Leakiness and creepiness in app space: perceptions of privacy and mobile app use.

[43] (2016). Regulation (EU) 2016/679 of the European Parliament and of the Council of 27 April 2016 on the protection of natural persons with regard to the processing of personal data and on the free movement of such data, and repealing Directive 95/46/EC (General Data Protection Regulation). https://eur-lex.europa.eu/legal-content/EN/ALL/?uri=celex%3A32016R0679.

[44] Kuner C (2012). The European Commission's proposed data protection regulation: A copernican revolution in European data protection law. Bloomberg BNA Privacy and Security Law Report.

[45] Zeevi D, Korem T, Zmora N, Israeli D, Rothschild D, Weinberger A, Ben-Yacov O, Lador D, Avnit-Sagi T, Lotan-Pompan M, Suez J, Mahdi JA, Matot E, Malka G, Kosower N, Rein M, Zilberman-Schapira G, Dohnalová Lenka, Pevsner-Fischer M, Bikovsky R, Halpern Z, Elinav E, Segal E (2015). Personalized Nutrition by Prediction of Glycemic Responses. Cell.

[46] Wang Y, Norice G, Cranor L F (2011). Who Is Concerned about What? A Study of American, Chinese and Indian Users’ Privacy Concerns on Social Network Sites.

[47] Krasnova H, Günther O, Spiekermann S, Koroleva K (2009). Privacy concerns and identity in online social networks. IDIS.

[48] Curmi F, Ferrario M A, Whittle Jon (2014). BioShare: a research tool for analyzing social networks effects when sharing biometric data. https://dl.acm.org/doi/10.1145/2598784.2602793.

[49] Woldaregay Ashenafi Zebene, Henriksen André, Issom David-Zacharie, Pfuhl Gerit, Sato Keiichi, Richard Aude, Lovis Christian, Årsand Eirik, Rochat Jessica, Hartvigsen Gunnar (2020). User Expectations and Willingness to Share Self-Collected Health Data. Stud Health Technol Inform.

[50] Cate FH (2002). Principles of Internet Privacy. Articles by Maurer Faculty.

