# Supplementary material for: Privacy Perceptions and Concerns in Image-Based Dietary Assessment Systems: Questionnaire-Based Study
Source: JMIR Hum Factors. 2020 Oct 15;7(4):e19085. doi: 10.2196/19085 (PMC7596657; doi:10.2196/19085)
Supplement: Multimedia Appendix 2 [file humanfactors_v7i4e19085_app2.zip › column_descriptions.pdf]

First part of the questionnaire

In case of lengthy response options, please note that the items marked in **bold** are the possible response options available in the dataset.

Reference	Question	Response options	Remarks, if any
a01	Do you use any social media like facebook, twitter, instagram, snapchat etc?	Yes / No	-
a02	Have you seen pictures of food (like the following) on social media?	Yes, **many** (>10) times/ Yes, a **few** times / Yes, **once**/ **No**	Only if response to a01 is 'Yes'
a03	Have you posted pictures of food over social media?	Yes, many (>10) times/ Yes, a few times / Yes, once/ No	-

Imagine yourself as an athlete representing an elite club in your country. Your contract with the club mandates that you take pictures of your dietary intake via an app, whenever you are eating. This includes breakfast, lunch and other meals including snacks

and drinks at events. **Even for private events when you are not training**, you are required to take pictures of your food intake. All this recording is done via a mobile application (app). The app allows your club owner, manager, coach and doctor to monitor your diet plan and offer recommendations. The app takes your individual food intake pictures and stores it online. For the following set of questions, indicate your response on a scale of strong disagreement to strong agreement.

5 point Likert scale : Strongly Disagree / Disagree / Neutral / Agree / Strongly Agree

Reference	Question	Response options	Remark if any
a04	Is it uncomfortable if someone learns about your everyday diet without your knowledge?	5 point Likert scale	-
a05	Is it intrusive to take pictures of food every time you eat?	5 point Likert scale	-
a06	Is it possible to protect privacy when posting content online?	5 point Likert scale	-
a07	Should the government protect privacy of its citizens?	5 point Likert scale	-
	Can posting pictures of your food lead to your	5 point	

a08	privacy being exposed online?	Likert scale	-
a09	Does posting food pictures online enhance one's reputation?	5 point Likert scale	-
a10	Does regularly posting food pictures online help one in maintaining a strict diet plan?	5 point Likert scale	-
a11	Is it ok if someone learns where you are eating everyday?	5 point Likert scale	-
a12	Is taking a picture of food easier than writing down what you ate?	5 point Likert scale	-
a13	Is taking a picture of food easier than recording audio describing what you ate?	5 point Likert scale	-
a14	Should first responders/paramedics have access to your dietary data for last week without your consent?	5 point Likert scale	-
	Is it harmless to share	5 point	

a15	food pictures on a regular basis?	Likert scale	-
a16	Should your immediate family members have access to your dietary data?	5 point Likert scale	-
a17	Is posting selfie(s) on social media good?	5 point Likert scale	-
a18	Should your fellow athletes have access to your dietary data?	5 point Likert scale	-
a19	Should anyone be able to look up when you ate food?	5 point Likert scale	-
a20	Can your team manager show your dietary data and analytics to other team members?	5 point Likert scale	-
a21	Should a notification be sent to you whenever someone uses your dietary data?	5 point Likert scale	-
a22	Should any use of your data require an explanation in simple clear words?	5 point Likert scale	-

a23	Can your anonymized data and analytics stay with your previous team, if you change team/club?	5 point Likert scale	-
a24	Does gender play a role in privacy concerns?	5 point Likert scale	-
a25	Can your doctor share your data for research with his/her colleagues without your consent?	5 point Likert scale	-

Second part of the questionnaire

Imagine a scenario in which you took picture of everything you consumed in last one year. The pictures only include images of food, including your prescribed medication, without your face. **The data did not include your name/email or identity**. Due to some reasons, a third-party (company/person) has gained access to your dietary data which includes images of the food you ate for last one year, along with location where it was taken, and timestamp when it was taken. The third-party has access to enormous computing infrastructure, employs machine learning methods and can use publicly available data from the internet.

In your opinion, do you think it is possible to figure out the following based on your diet data.

Reference	Question	Response options	Remarks, if any
b01	Allergies	Not likely/ Somewhat likely/ Very likely	-
b02	Religion	Not likely/ Somewhat likely/ Very likely	-
b03	Nationality	Not likely/ Somewhat likely/ Very likely	-
b04	Economical	Not likely/ Somewhat likely/	-

	status	Very likely	
b05	Health status	Not likely/ Somewhat likely/ Very likely	-
b06	Identity	Not likely/ Somewhat likely/ Very likely	-

Out of the above factors, imagine someone is able to learn all of them. How concerned do you feel about someone (third party/ business) learning about your

In case of lengthy response options, please note that the items marked in **bold** are the possible response options available in the dataset.

Reference	Question	Response options	Remarks, if any
b07	Allergies	Not concerned/ Somewhat concerned/ Very concerned	-
b08	Religion	Not concerned/ Somewhat concerned/ Very	-

		concerned	
b09	Nationality	Not concerned/ Somewhat concerned/ Very concerned	-
b10	Economical status	Not concerned/ Somewhat concerned/ Very concerned	-
b11	Health status	Not concerned/ Somewhat concerned/ Very concerned	-
b12	Identity	Not concerned/ Somewhat concerned/ Very concerned	-
	In case a doctor needs	Last **24 hours**/ **3**	*Dietary intake is the food that you ate.*

b13	to look at your dietary intake, how far back in time should he/she have access?	**days**/ **week**/ **2 weeks**/ **month**/ **All** of it	*This might be recorded in terms of descriptive text, or food pictures.*
b14	Should your family members have access to your dietary intake?	**Yes**/ Only, if I **explicitly** say so/ Only in case of **emergency**/ **No**	-

Third part of the the questionnaire

For the following series of items, choose whichever person/groups you are comfortable sharing the listed items. For example, if you are comfortable sharing "Information about your daily breakfast" with ONLY your **family** and **friends**, only those should be marked.

In the dataset, marked is indicated by the value **1**. Empty cells indicate, unmarked.

	**Family (Reference)**	**Friends (Reference)**	**Docto (Referen**
Pictures/information about your daily diet	[ ] c1a	[ ] c1b	[ ] c1c
Pictures/information about your weight/BMI	[ ] c2a	[ ] c2b	[ ] c2c
Pictures/information about your consumption of alcohol	[ ] c3a	[ ] c3b	[ ] c3c
Pictures/information about your consumption of over the counter medication	[ ] c4a	[ ] c4b	[ ] c4c
Pictures/information			

about your consumption of prescription medication	[ ] c5a	[ ] c5b	[ ] c5[illegible]
Pictures/information about your consumption of narcotic drugs, given that there is **NO LEGAL PERSECUTION**	[ ] c6a	[ ] c6b	[ ] c6[illegible]
Your health report and diet plan	[ ] c7a	[ ] c7b	[ ] c7[illegible]
Location of places where you eat	[ ] c8a	[ ] c8b	[ ] c8[illegible]
People you eat your food with	[ ] c9a	[ ] c9b	[ ] c9[illegible]
Time when you eat your food	[ ] c10a	[ ] c10b	[ ] c10[illegible]

Fourth part of the the questionnaire

In case of lengthy response options, please note that the items marked in **bold** are the possible response options available in the dataset.

Reference	Question	Response options	Remarks, if any
d01	What is your country of origin	List of countries	*Please select the country you are from. In case you hold multiple nationalities, please select the one you prefer.*
d02	How old are you?	18-25/ 25-35/ 35-45/ 45-55/ 55-65/ 65+	Select your age group
d03	What is your gender?	Male/ Female/ Non-binary	*optional*
d04	What is your educational qualification?	**Primary** School/ **Middle** School/ **High** School/ **Diploma** / **Bachelors**	Choose your highest/curren[illegible] degree

		Degree/ **Professional** Certification / **Postgraduate** / **Doctorate**	program
d05	Are you aware of any privacy laws that are applicable to your region?	**Yes**/ Only their **names**/ A **little** / **No**	For example, European Union has GDPR, Data Protection Directive (defunct), USA has COPPA, HIPAA etc.
d06	Do you follow a religious diet	Yes/ No	*optional*
d07	Do you have any allergies	Yes/ No	*optional*
