# Supplementary material for: Privacy Perceptions and Concerns in Image-Based Dietary Assessment Systems: Questionnaire-Based Study
Source: JMIR Hum Factors. 2020 Oct 15;7(4):e19085. doi: 10.2196/19085 (PMC7596657; doi:10.2196/19085)
Supplement: Multimedia Appendix 1 [file humanfactors_v7i4e19085_app1.pdf]

Privacy Perception Survey (Photographic Food Diary)
Change title

Open for submissions?
The form is open
Closed

Last modified
9. september 2019 14:40
by Aakash Sharma

View | Form builder | Settings | Permissions | Collect responses | See results

Privacy Perception Survey (Photographic Food Diary)

Page 1

Purpose

This survey is made by the University of Tromsø as a part of a research project that aims to better understand how individuals think about privacy.

No personally identifiable information will be collected during the survey. This survey will only use the collected data for research purposes. The data will not be shared with any third parties, inside or outside the University of Tromsø.

For more information about the purpose of the survey and the analysis of the collected data, contact Aakash (aakash.sharma@uit.no, +47 94428984).

To disseminate collected information to researchers in a responsible manner, machine learning methods will be used to understand underlying behavior. Depending upon the accuracy of the model, it might be made available to other researchers. The model will NOT contain any raw data. It is a mathematical representation of the aggregate data.

Page break

Page 2

Do you use any social media like facebook, twitter, instagram, snapchat etc? *

- ○ Yes
- ○ No

Have you seen pictures of food (like the following) on social media? *

This element is only shown when the option "Yes" is selected in the question "Do you use any social media like facebook, twitter, instagram, snapchat etc?"

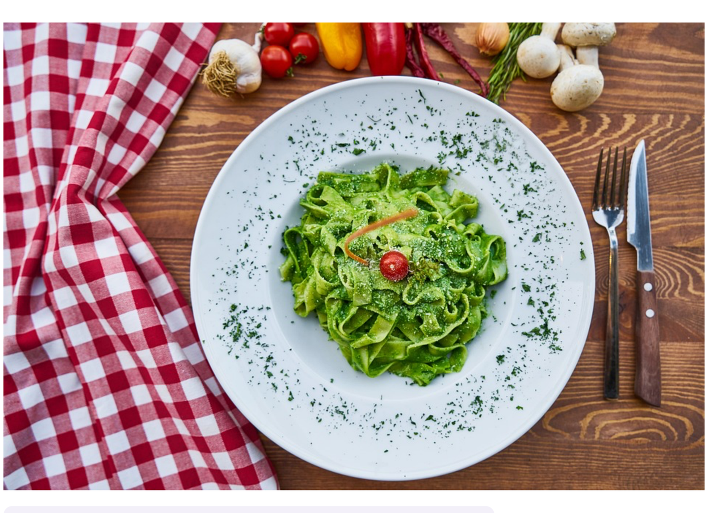

- ○ Yes, many (>10) times
- ○ Yes, a few times
- ○ Yes, once
- ○ No, never

Have you posted pictures of food over social media? *

- ○ Yes, many (>10) times
- ○ Yes, a few times
- ○ Yes, once
- ○ No, never

Page break

Page 3

Imagine yourself as an athlete representing an elite club in your country. Your contract with the club mandates that you take pictures of your dietary intake via an app, whenever you are eating. This includes breakfast, lunch and other meals including snacks and drinks at events. **Even for private events when you are not training**, you are required to take pictures of your food intake. All this recording is done via a mobile application (app). The app allows your club owner, manager, coach and doctor to monitor your diet plan and offer recommendations. The app takes your individual food intake pictures and stores it online.

For the following set of questions, indicate your response on a scale of strong diagreement to strong agreement.

	Strongly Disagree	Disagree	Undecided	Agree	Strongly Agree
Is it uncomfortable if someone learns about your everyday diet without your knowledge? *	○	○	○	○	○
Is it intrusive to take pictures of food every time you eat? *	○	○	○	○	○
Is it possible to protect privacy when posting content online? *	○	○	○	○	○
Should the government protect privacy of its citizens? *	○	○	○	○	○
Can posting pictures of your food lead to your privacy being exposed online? *	○	○	○	○	○
Does posting food pictures online enhance one's reputation? *	○	○	○	○	○
Does regularly posting food pictures online help one in maintaining a strict diet plan? *	○	○	○	○	○
Is it ok if someone learns where you are eating everyday? *	○	○	○	○	○
Is taking a picture of food easier than writing down what you ate? *	○	○	○	○	○
Is taking a picture of food easier than recording audio describing what you ate? *	○	○	○	○	○
Should first responders/paramedics have access to your dietary data for last week without your consent? *	○	○	○	○	○
Is it harmless to share food pictures on a regular basis? *	○	○	○	○	○
Should your immediate family members have access to your dietary data? *	○	○	○	○	○
Is posting selfie(s) on social media good? *	○	○	○	○	○
Should your fellow athletes have access to your dietary data? *	○	○	○	○	○
Should anyone be able to look up when you ate food? *	○	○	○	○	○
Can your team manager show your dietary data and analytics to other team members? *	○	○	○	○	○
Should a notification be sent to you whenever someone uses your dietary data? *	○	○	○	○	○
Should any use of your data require an explanation in simple clear words? *	○	○	○	○	○
Can your anonymized data and analytics stay with your previous team, if you change team/club? *	○	○	○	○	○
Does gender play a role in privacy concerns? *	○	○	○	○	○
Can your doctor share your data for research with his/her colleagues without your consent? *	○	○	○	○	○

Page break

Page 4

Imagine a scenario in which you took picture of everything you consumed in last one year. The pictures only include images of food, including your prescribed medication, without your face. **The data did not include your name/email or identity.** Due to some reasons, a third-party (company/person) has gained access to your dietary data which includes images of the food you ate for last one year, along with location where it was taken, and timestamp when it was taken. The third-party has access to enormous computing infrastructure, employs machine learning methods and can use publicly available data from the internet.

In your opinion, do you think it is possible to figure out the following based on your diet data.

	Not likely	Somewhat likely	Very likely
Allergies? *	○	○	○
Religion? *	○	○	○
Nationality? *	○	○	○
Economical status? *	○	○	○
Health status? *	○	○	○
Identity? *	○	○	○

Out of the above factors, imagine someone is able to learn all of them. How concerned do you feel about someone (third party/ business) learning about your .

	Not Concerned	Somewhat Concerned	Very Concerned
Allergies? *	○	○	○
Religion? *	○	○	○
Nationality? *	○	○	○
Economical Status? *	○	○	○
Health Status? *	○	○	○
Identity? *	○	○	○

Page break

Page 5

In case a doctor needs to look at your dietary intake, how far back in time should he/she have access? *

Dietary intake is the food that you ate. This might be recorded in terms of descriptive text, or food pictures.

- ○ Last 24 hours
- ○ Last 3 days
- ○ Last week
- ○ Last 2 weeks
- ○ Last month
- ○ All of it

Should your family members have access to your dietary intake? *

Dietary intake is the food that you ate. This might be recorded in terms of descriptive text, or food pictures.

- ○ Yes
- ○ Only, if I explicitly say so
- ○ Only in case of emergency
- ○ No

For the following series of items, choose whichever person/groups you are comfortable sharing the listed items. For example, if you are comfortable sharing "Information about your daily breakfast" with ONLY your **family** and **close friends**, only those should be marked.

For faster response, we have pre-selected Family and your doctor.

	Family	Close Friends	Your doctor	Your team members	Your fans / followers	Not with anyone
Pictures/information about your daily diet *	☑	☐	☑	☐	☐	☐
Pictures/information about your weight/BMI *	☑	☐	☑	☐	☐	☐
Pictures/information about your consumption of alcohol *	☑	☐	☑	☐	☐	☐
Pictures/information about your consumption of over the counter medication	☑	☐	☑	☐	☐	☐
Pictures/information about your consumption of prescription medication *	☑	☐	☑	☐	☐	☐
Pictures/information about your consumption of narcotic drugs, given that there is NO LEGAL PERSECUTION	☑	☐	☑	☐	☐	☐
Your health report and diet plan *	☑	☐	☑	☐	☐	☐
Location of places where you eat *	☑	☐	☑	☐	☐	☐
People you eat your food with *	☑	☐	☑	☐	☐	☐
Time when you eat your food *	☑	☐	☑	☐	☐	☐

Page break

Page 6

General questions about you

What is your country of origin? *

Please select the country you are from. In case you hold multiple nationalities, please select the one you prefer.

Select ...

How old are you? *

Select your age group

Select ...

What is your gender?

- ○ Male
- ○ Female
- ○ Non-binary

What is your educational qualification? *

Choose your highest/current degree program.

Select ...

Are you aware of any privacy laws that are applicable to your region? *

For example, European Union has GDPR, Data Protection Directive (defunct), USA has COPPA, HIPAA etc.

- ○ Yes
- ○ Only their names
- ○ A little
- ○ No

Do you follow a religious diet?

- ○ Yes
- ○ No

Do you have any food allergies?

- ○ Yes
- ○ No

Any other comment or general feedback?

Feel free to write any comment or concerns you might have about this survey. We promise to keep it confidential as we are not storing any identifiable information about you.
